# Integrated primary and secondary care optimizes the management of people with CKD—the LUCID project

**DOI:** 10.1093/ckj/sfaf049

**Published:** 2025-02-12

**Authors:** Rupert W Major, Niraj Lakhani, Yaseen Ahmed, Jade Atkin, Richard Baines, Rose Balment, Chee Kay Cheung, Matthew P Graham-Brown, Claire Ellwood, Laura Harding, Osasuyi Iyasere, Tracy Jesa, Jorge Jesus-Silva, Yusuf Jinadu, Arshad Khalid, Jibran Khatri, Yahya Makkeyah, Maria Martinez, Helen Mather, James F Medcalf, Kirk Moore, James Ogle, Eleanor Oseya, Dipesh Patel, Reena Patel, Tracy Pollard, William Priestman, Amit Rastogi, Nil Sanganee, Mark Shaffu, Michael Steiner, Tun Than, Gang Xu, Fahad Rizvi, James O Burton

**Affiliations:** Department of Population Health Sciences, College of Life Sciences, University of Leicester, Leicester, UK; University of Hospitals of Leicester NHS Trust, Leicester, UK; Willows Health Primary Care Network, Leicester, UK; University of Hospitals of Leicester NHS Trust, Leicester, UK; North West Leicestershire Primary Care Network, Leicestershire, UK; Leicester, Leicestershire and Rutland Integrated Care Board, Leicester, UK; University of Hospitals of Leicester NHS Trust, Leicester, UK; North West Leicestershire Primary Care Network, Leicestershire, UK; University of Hospitals of Leicester NHS Trust, Leicester, UK; Department of Cardiovascular Sciences, College of Life Sciences, University of Leicester, Leicester, UK; University of Hospitals of Leicester NHS Trust, Leicester, UK; Department of Cardiovascular Sciences, College of Life Sciences, University of Leicester, Leicester, UK; NIHR Leicester Biomedical Research Centre, University of Leicester, Leicester, UK; Leicester, Leicestershire and Rutland Integrated Care Board, Leicester, UK; North West Leicestershire Primary Care Network, Leicestershire, UK; University of Hospitals of Leicester NHS Trust, Leicester, UK; Department of Cardiovascular Sciences, College of Life Sciences, University of Leicester, Leicester, UK; Leicester, Leicestershire and Rutland Integrated Care Board, Leicester, UK; University of Hospitals of Leicester NHS Trust, Leicester, UK; University of Hospitals of Leicester NHS Trust, Leicester, UK; Fosseway Primary Care Network, Leicestershire, UK; University of Hospitals of Leicester NHS Trust, Leicester, UK; University of Hospitals of Leicester NHS Trust, Leicester, UK; University of Hospitals of Leicester NHS Trust, Leicester, UK; Leicester, Leicestershire and Rutland Integrated Care Board, Leicester, UK; University of Hospitals of Leicester NHS Trust, Leicester, UK; Department of Cardiovascular Sciences, College of Life Sciences, University of Leicester, Leicester, UK; North West Leicestershire Primary Care Network, Leicestershire, UK; Bosworth Primary Care Network, Leicestershire, UK; Leicester City South Primary Care Network, Leicester, UK; University of Hospitals of Leicester NHS Trust, Leicester, UK; Belgrave and Spinney Hill Primary Care Network, Leicester, UK; Leicester City South Primary Care Network, Leicester, UK; Bosworth Primary Care Network, Leicestershire, UK; Leicester City South Primary Care Network, Leicester, UK; Leicester, Leicestershire and Rutland Integrated Care Board, Leicester, UK; Oadby and Wigston Primary Care Network, Leicester, UK; University of Hospitals of Leicester NHS Trust, Leicester, UK; Leicester, Leicestershire and Rutland Integrated Care Board, Leicester, UK; Department of Respiratory Sciences, College of Life Sciences, University of Leicester, Leicester, UK; Salutem Primary Care Network, Leicester, UK; University of Hospitals of Leicester NHS Trust, Leicester, UK; Willows Health Primary Care Network, Leicester, UK; Leicester, Leicestershire and Rutland Integrated Care Board, Leicester, UK; University of Hospitals of Leicester NHS Trust, Leicester, UK; Department of Cardiovascular Sciences, College of Life Sciences, University of Leicester, Leicester, UK; NIHR Leicester Biomedical Research Centre, University of Leicester, Leicester, UK

**Keywords:** CKD, CVD, integrated care, KFRE, medicine optimisation

## Abstract

**Background:**

Early diagnosis, risk stratification and medication optimization are essential to improve the management of chronic kidney disease (CKD) and other long-term conditions. The introduction of Integrated Care Systems (ICS) in England provides the opportunity to revolutionize the management of these conditions. Annual National Health Service kidney disease costs are ∼£6.4 billion.

**Methods:**

We designed, piloted and implemented at scale an ICS-level virtual care programme for CKD, the ‘Leicester, Leicestershire, and Rutland Chronic Kidney Disease Integrated Care Delivery Project’ (LUCID), based on the principles of patient and professional education, early disease identification, medicines optimization and disease surveillance.

**Results:**

In April 2022, virtual multidisciplinary team (MDT) meetings were piloted in Leicester, Leicestershire and Rutland, UK. Since April 2023 virtual MDT meetings have been available to all general practices in Leicester, Leicestershire and Rutland, representing a population of approximately 1.2 million people. As of 31 March 2024, general practices representing an estimated population of 700 000 (58.3%) were participating in the LUCID programme. Some 1085 consultations took place for 821 patients, 590 (54.4%) of which were medicines optimization consultations.

**Conclusions:**

LUCID may represent an efficient and cost-effective model to deliver patient and professional education, medicine optimization and risk stratification for people living with CKD at an ICS-wide population level. This model may be adaptable for other long-term physical and mental health conditions.

KEY LEARNING POINTS **What was known:**

- Early diagnosis, risk stratification and medication optimization are essential to improve the management of chronic kidney disease (CKD) and other long-term conditions.
- In England, Integrated Care Systems (ICS) present an opportunity to tackle these issues at a population level.

**This study adds:**

- LUCID integrated virtual multidisciplinary team meetings for CKD led to medication optimization of 590 people living with kidney disease, many of whom had other cardiovascular comorbidities.

**Potential impact:**

- LUCID may represent an efficient and cost-effective model to deliver improved patient and professional educational awareness, medicine optimization and risk stratification for people living with CKD at an ICS-wide population level.
- The LUCID model may be applicable to other long-term physical and mental health conditions, but further work is required to assess this.

## INTRODUCTION

Early diagnosis, risk stratification and increased use of evidence-based medications are essential to improve outcomes for patients with chronic kidney disease (CKD) [1]. Increased rates of urine proteinuria measurement, use of the Kidney Failure Risk Equation (KFRE) [2, 3] and systematic optimization of evidence-based pharmacotherapies have been identified by The National Institute for Health and Care Excellence (NICE) as the cornerstones of management in CKD [1, 4–6]. The vast majority of people with CKD are managed in a traditional primary care setting. In England, the introduction of Integrated Care Systems (ICS)—which allocate responsibility for population health across primary and secondary care—provides the opportunity to revolutionize CKD care and other chronic disease management by focusing on population health [7]. However, barriers to implementation remain including unprecedented demands on services, data integration across systems and differences in organizational and professional cultures [7].

An estimated 7.2 million people in the UK are thought to be currently living with CKD [8]. Globally, kidney disease is forecast to be the fifth leading cause of premature death by 2040 [9]. CKD is associated with a reduction in health-related quality of life and leads to premature death for thousands of people [8, 9].

Approximately 35 000 people in the UK require some form of kidney replacement therapy (KRT) in the form of dialysis or kidney transplantation [10]. Due to the number of comorbidities experienced by people with advanced CKD, and the lack of availability of organs for transplantation, dialysis remains the only option for most, at a cost of £35 000 per person per year [8]. Currently approximately £6.4 billion, or 3.2% of the budget of the National Health Service (NHS) is spent on kidney disease, but an independent report published by Kidney Research UK highlighted that KRT need could reach 143 000 individuals, at a cost to the NHS of £13.9 billion, by 2033 [8].

People with CKD have an increased risk of cardiovascular events, with an associated excess cost of more than £250 million per year for NHS England [11]. Prolonged acute hospital admissions for people with CKD cost the NHS an estimated additional £70 million due to excess bed days [11].

Improving outcomes for people living with CKD remains a challenge. Historically, limited evidence that outcomes could be modified by therapeutic interventions led to clinical inertia [12]. However, recent developments in the management of CKD and related cardiometabolic diseases have provided hope that outcomes could be improved for people living with kidney disease [13–16]. The updated NICE CKD guidelines in 2021 included the use of the KFRE and sodium-glucose cotransporter 2 (SGLT2) inhibitors to improve individualized and population level care for CKD and the risk of both progression to KRT and cardiovascular events [1]. A previous clinical trial for CKD has demonstrated that systems processes such as CKD coding and proteinuria measurement may be improved by more collaborative working between primary and secondary care [17].

There are 42 ICS across England with the remit to improve population health and healthcare [7]. This includes decentralization of decision making to ensure the needs of the local population are met. Leicester, Leicestershire and Rutland (LLR) ICS is located in the East Midlands of England and is responsible for the health of approximately 1.2 million people. ICS are further divided into Primary Care Networks (PCN) which are group of general practices serving approximately 50 000 people to support improved collaboration between practices for services whilst still providing health and social care in the community.

LLR ICS has many people living with health inequalities due to socioeconomic deprivation, ethnicity (including a non-English first language) and multiple long-term conditions, all of which are associated with adverse outcomes for kidney disease, including late diagnosis, low uptake of evidence-based therapies, higher rates of cardiovascular events and increased need for KRT [17].

We implemented an integrated programme for CKD involving collaboration between primary and secondary care aimed at delivering effective evidence-based care for people living with CKD to improve outcomes at a population level. The establishment of ICS offers the organizational landscape for this to happen but most importantly to develop, scale and extend the model into other chronic disease areas.

This manuscript describes the delivery of the LUCID intervention at a healthcare system level. Results from one of the pilot PCN's quality improvement programme for CKD and have been previously presented by Rizvi *et al.* [18].

## MATERIALS AND METHODS

We designed, piloted and implemented at scale an ICS-level virtual programme for CKD, the ‘Leicester, Leicestershire, and Rutland Chronic Kidney Disease Integrated Care Delivery Project’ (LUCID). Rizvi *et al.* describes the intervention for one pilot PCN in detail, but as a brief summary, LUCID is based on the principles of:

(i) Patient and professional education
(ii) Early disease identification
(iii) Medicines optimization
(iv) Disease surveillance

**Figure 1:**
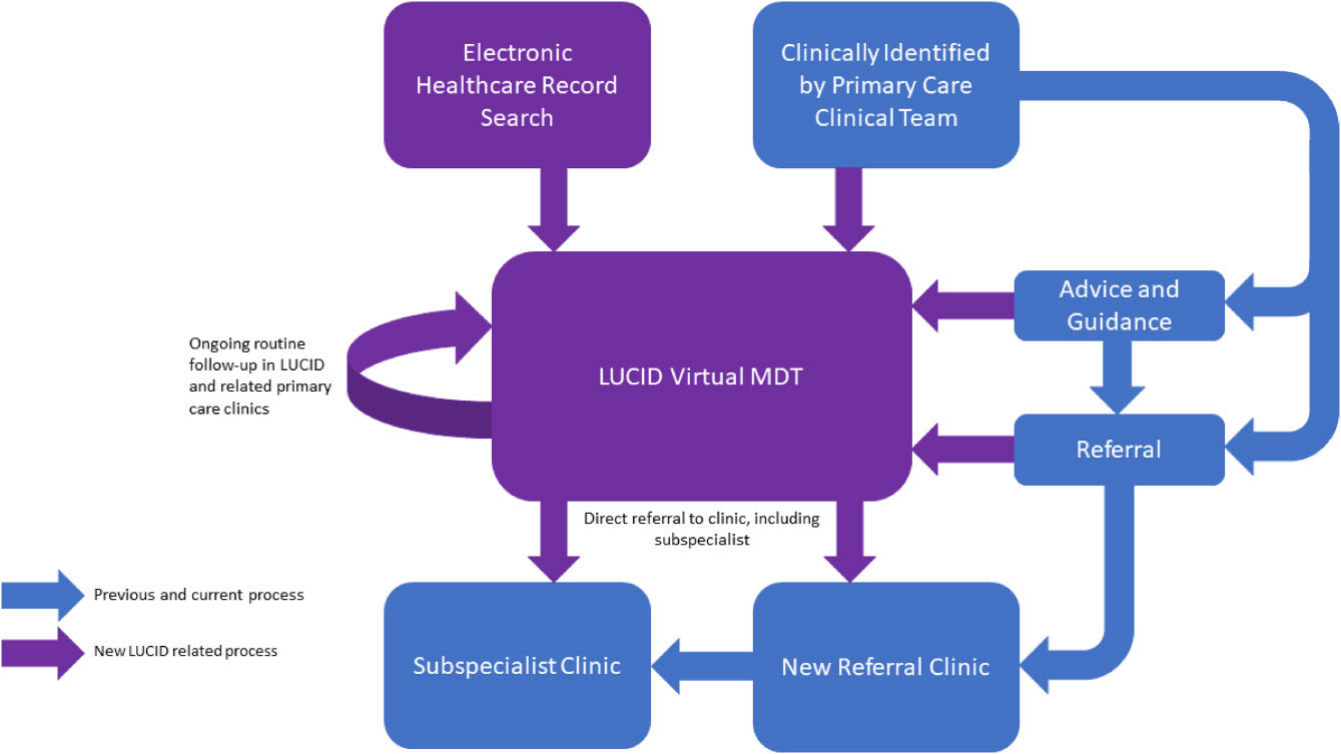
Flowchart demonstrating change to clinical processes related to LUCID. Blue, existing and ongoing process; purple, new processes related to LUCID.

We developed LUCID using the principles of quality improvement through patient and professional education and delivery of virtual multidisciplinary team (MDT) meetings. The term ‘virtual’ is defined as ‘done using computer technology over the internet, and not involving people physically going somewhere’ [19], throughout the manuscript. A comparison of the change in work flow is shown in Fig. 1. Programme development began in 2021 focusing on the public kidney education programme of educational videos, to date these videos have been viewed 51 000 times [20] Professional education sessions have been delivered at ICS-wide events and to 18 out of the 26 PCN using standardized teaching materials. Both the patient and professional education programmes were developed using an informal feedback-driven iterative approach from users and participants of the material. No formal evaluation of this process was undertaken.

Patient identification was supported through the use of electronic healthcare records searches (Apollo Medical Software Solutions Ltd, UK and supported by AstraZeneca, UK) performed at PCN level, including risk stratification using the KFRE and criteria for guideline-directed medical therapies. All MDT meetings were virtual and performed using Microsoft Teams, composed of a consultant nephrologist and a combination of specialist pharmacist, primary care pharmacist, practice nurse and/or primary care physician. The public education videos supported medicine optimization and were made available during consultations or through an internet link within the medical record that the patient was able to access.

Outcomes of the MDT reviews, including potential options available, were discussed with the patient by their primary care teams.

MDT meeting level data were collected as part of the service evaluation based on the following outcome definitions:

(i) ‘Medicines optimized’—a change in medications in line with guideline-based interventions. This included lipid lowering medicines management, blood pressure medication titration (including use of angiotensin-converting enzyme inhibitors and angiotensin-receptor blockers) and SGLT2 inhibitor initiation based on NICE guidelines.
(ii) ‘Referral avoided’ and ‘Advice and guidance avoided’—based on risk-based triage of individuals using the NICE recommended KFRE and, where appropriate, management in primary care within LUCID clinics. For these outcomes, in the absence of the MDT then either a referral or an ‘Advice and guidance’ request would have been made.
(iii) ‘Expedited referrals’—where high risk patients had not been referred for outpatient review and were thought to be requiring review, review was fast tracked either to a standard secondary care nephrology care outpatient or specialist clinic. For this outcome, in the absence of the MDT, the patient would have not been referred to secondary.

PCN were able to organize the virtual clinics in their preferred format with no strict criteria for ‘referral’ into the clinics. The use of the electronic healthcare record–based audit tools was at the discretion of individual practices and secondary care physicians did not directly review the identifiable content of the dashboard. All PCN clinics involved a feedback process either during the clinics or at the end through a ‘debriefing’ session depending on the wishes of the PCN. Professional education sessions were provided at a PCN level to all PCN and case-based learning was central to all clinics and debriefing sessions.

An external evaluation of LUCID was commissioned by Health Innovation East Midlands and performed by Edge Health Limited (copy available on request to the authors).

## RESULTS

**Figure 2:**
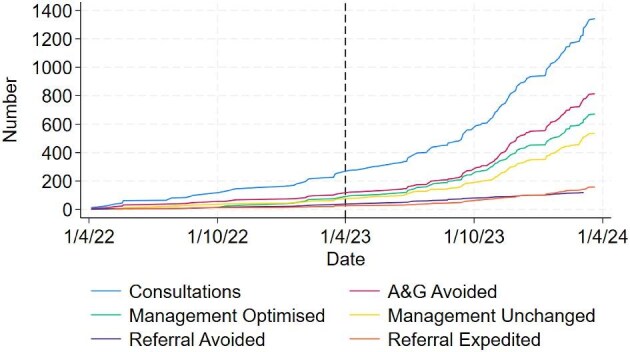
Cumulative outcomes for the LUCID project. Vertical black dotted line represents the beginning of the full programme. A&G, Advice and guidance.

In April 2022 virtual clinics were piloted in four PCN and since April 2023 have been available to all 26 PCN. As of 31 March 2024, 15 out of 26 PCN (57.7%), representing primary care service for an estimated population of 700 000 (58.3%), are participating in the programme (Table 1). Between 1 April 2023 and 31 March 2024, 1085 virtual patient discussions took place for 821 patients. A total of 590 (54.4%) consultations involved medicines optimization, 84 (7.7%) avoiding a referral to secondary care and 132 (12.2%) leading to expedited secondary care. Figure 2 shows consultation types by date for the pilot and full programme.

**Table 1:** Descriptive summary of LUCID pilot and full programme.

		Full programme		
	Pilot	All consultations	1st consultation	Follow-up
PCN, *n*	4	15		
Consultations, *n*	256	1085	821	264
Clinics, *n*	21	102		
Consultations per clinic				
Mean (SD)	12.4 (6.3)	10.3 (7.5)		
Median (IQR)	13 (7 to 18)	8 (5 to 16)		
Comorbidities, *n* (%)				
Hypertension		812 (74.8)	623 (75.9)	189 (71.6)
Diabetes mellitus		602 (55.5)	463 (56.4)	139 (52.7)
Heart failure		154 (14.2)	128 (15.6)	26 (9.9)
Outcomes, *n* (%)				
Medicines optimization	81	590 (54.4)	510 (62.1)	80 (30.3)
Already optimized	73	462 (42.6)	290 (35.3)	172 (65.2)
Referrals avoided	36	84 (7.7)	72 (8.8)	12 (4.6)
Referrals expedited	26	132 (12.2)	112 (13.6)	20 (7.6)

Pilot refers to 1 April 2022 to 31 March 2023 and Full Programme 1 April 2034 to 31 March 2024. Data for comorbidities were not collected during pilot.

SD, standard deviation; IQR, interquartile range.

### Estimated long-term costs and benefits per PCN

A total of 102 clinics were completed between 1 April 2022 and 31 March 2024 with multi-professional input into each clinic from a primary care physician, practice nurse and/or pharmacist with a consultant nephrologist and specialist pharmacist. Table 2 shows the breakdown of the estimated annual cost of £7948 of delivering one clinic per month per PCN, excluding administrative support and time. Accounting for rising primary care clinical time and costs from increased appropriate diagnosis of CKD, this increased to £9523 per PCN by Year 3 (Table 2). This did not account for additional time outside of the clinic to discuss medicines optimization directly with the patient. Clinics were assumed to have a consultant nephrologist present throughout with intermittent input by primary care representatives and support from a specialist secondary care kidney pharmacist.

**Table 2:** Estimated annual costs per PCN for staffing costs related to clinics.

				Costs		
PCN costs	NHS band	WTE	Hours/week	Year 1	Year 2	Year 3
Primary care						
General practitioner	N/A	0.007	0.28	800	1000	1250
Pharmacists	8a	0.016	0.64	1076	1345	1681
Nurse practitioners	7	0.016	0.64	924	1155	1444
Total primary care costs				£2800	£3500	£4375
Secondary care						
Consultant nephrologist	N/A	0.036	1.44	4370	4370	4370
Specialist pharmacist	8b	0.020	0.80	778	778	778
Total secondary care costs				£5148	£5148	£5148
Annual cost per PCN				£7948	£8648	£9523

WTE: whole time equivalent; N/A: non-applicable.

Total costs include uplift for other staff-related costs. Third year costs account for a 25% uplift in years two and three of primary care costs related to potential increase in CKD case finding in relation to the programme. Cost at 2022/23 values and do not account for future inflation.

### External evaluation

An external, independent evaluation of the project was performed (Edge Health, London, UK) using data available to 21 September 2023 and is available on request from the authors.

## DISCUSSION

The LUCID programme has demonstrated that an integrated programme of CKD care involving close collaboration between primary and secondary care clinical teams can improve the delivery of evidence-based care for people living with CKD. The increasing prevalence of long-term conditions has, and will continue to have, a major impact on quality and quantity of life and economic costs for healthcare systems worldwide [9]. The increasing complexity of healthcare, particularly in the setting of multiple long-term conditions, has increased the propensity of secondary care towards working in specialist silos without a holistic overview of a patient. Primary care has traditionally held this role for patients and their carers. ICS provide the opportunity to bring these two seemingly disparate themes together for people living with long-term conditions by focusing on population health, earlier diagnoses and providing care in the patient's own ‘neighbourhood’ [7].

CKD and the often-related cardiometabolic conditions have profound impact on quantity and quality of life, with costs estimated to rise to at least £7 billion per year for the UK [8]. Approaches to deliver evidence-based early interventions at scale in a real-world setting have been limited. ICS may be able to support systems to allow early diagnosis, risk stratification and evidence-based medicine optimization, primarily for CKD but with impact for related cardiometabolic conditions.

The LUCID pilot tested this hypothesis prior to scaling of the intervention to all PCN within the LLR ICS. The associated initial cost per PCN of less than £10 000 led to short-term reduction in referrals and related administration time. We did not collect data to assess at a PCN level the potential impact of increased renin–angiotensin–aldosterone system inhibtor use, lipid-lowering medications and blood pressure medications.

Of note, approximately twice as many consultations took place in the second half of the full programme compared with the first half which may represent the lead in time required to establish and embed virtual clinics within a PCN.

Assessment of other interventions, such as those related to exercise and lifestyle advice were not systematically delivered or recorded. Future work within LUCID will include implementation studies of MyKidneys&Me, which has recently been shown in a clinical trial setting to improve patient activation [21]. In addition, specific interventions for hyperkalaemia management, which may have impacted on accident and emergency attendances for remeasurement and management, were not recorded.

Our work does have limitations. This was not a clinical trial of the service and we rely on observational data, routinely collected within the service by those performing the clinics. For the same reason we lack granularity within the collected data to segment medicine optimization impact. CKD interventions have been trialled within pragmatic trials with mixed results [17, 22]. We have no formal qualitative data regarding the impact of LUCID, but the good uptake by PCN and education focus of the programme may reflect positively in relation to this. Future work will assess the impact of the LUCID approach on patients’ views of how specialist care is delivered, especially how this impact concordance with specialist recommended interventions.

The LUCID intervention provides a model of care that could be extended to other conditions such as cardiometabolic, respiratory and mental health conditions. Additionally, future work will focus on non-pharmacological interventions such as lifestyle and exercise interventions. There are financial and contractual barriers to further scaling such as; moving resources from secondary to primary care, developing a contractual model that incentivizes NHS Trusts to deploy consultant resource away from secondary care, and recognizing the pressured finances and workload of primary care to deliver the virtual clinics in a long-term, sustainable manner.

## CONCLUSIONS

LUCID may represent an efficient model to deliver improved patient and professional educational awareness, medicine optimization and risk stratification for people living with CKD within at an ICS-wide population level. The model may be applicable to other long-term conditions and further work is required to assess this. This will start to address the major challenges healthcare systems face of people living with multiple long-term conditions and the related impact on quality and quantity of life.

## Data Availability

The data underlying this article will be shared on reasonable request to the corresponding author.
